# Myositis Autoantibodies in Patients with Suspected Post-Treatment Lyme Disease Syndrome

**DOI:** 10.3390/life13020527

**Published:** 2023-02-15

**Authors:** Kristyna Sloupenska, Barbora Koubkova, Pavel Horak, Beata Hutyrova, Mojmir Racansky, Jan Mares, Martina Miklusova, Jan Schovanek, Jana Zapletalova, Milan Raska, Michal Krupka

**Affiliations:** 1 Department of Immunology, Faculty of Medicine and Dentistry, Palacky University Olomouc, Hnevotinska 3, 779 00 Olomouc, Czech Republic; 2 Department of Allergology and Clinical Immunology, University Hospital Olomouc, Zdravotniku 248/7, 779 00 Olomouc, Czech Republic; 3 Third Department of Internal Medicine-Nephrology, Rheumatology and Endocrinology, University Hospital Olomouc, Zdravotniku 248/7, 779 00 Olomouc, Czech Republic; 4 Department of Neurology, University Hospital Olomouc, Zdravotniku 248/7, 779 00 Olomouc, Czech Republic; 5 Department of Medical Biophysics, Faculty of Medicine and Dentistry, Palacky University Olomouc, Hnevotinska 3, 779 00 Olomouc, Czech Republic; 6 Department of Immunology, University Hospital Olomouc, Zdravotniku 248/7, 779 00 Olomouc, Czech Republic

**Keywords:** Lyme disease, post-treatment Lyme disease syndrome, myositis, autoantibodies

## Abstract

Most patients suffering from Lyme disease are effectively treated with antibiotics. In some patients, however, problems persist for a long time despite appropriate therapy. The term post-treatment Lyme disease syndrome (PTLDS) is currently used for this condition in scientific literature. The pathogenesis is still not precisely known, but the involvement of immunopathological mechanisms is assumed. In our study, we analyzed the presence of autoantibodies including myositis-specific (MSA) and myositis-associated autoantibodies (MAA) in patients with laboratory proven history of Lyme disease and with clinical symptoms of PTLDS. A total of 59 patients meeting the criteria for PTLDS were enrolled in this study. The control group consisted of 40 patients undergoing differential diagnosis of neurological disorders without clinical and/or laboratory-proven history of Lyme disease. The presence of autoantibodies was determined by immunoblot methods and positive samples were further tested for serum creatine kinase (CK) and myoglobin levels. The presence of myositis autoantibodies was detected in 18 subjects with suspected PTLDS (30.5%), but only in 5% of control subjects exhibiting no evidence of Lyme disease history. The difference was statistically significant (*p* = 0.002). The subsequent biochemical analysis of muscle-damage markers in positive subjects found a mild elevation in six MSA/MAA-positive PTLDS patients. The study detected raised MSA/MAA autoantibodies formation in the group of PTLDS patients raising the question about their involvement in the pathogenesis of this syndrome.

## 1. Introduction

Lyme disease is the most frequent arthropod-borne infectious disease in humans in the Northern Hemisphere. It is a multi-system disorder with a diverse spectrum of clinical manifestations, caused by spirochetes of the *Borrelia burgdorferi* sensu lato (s. l.) complex that are transmitted by the bite of ticks of the genus *Ixodes* [1,2,3]. The *Borrelia burgdorferi* s. l. complex contains about 20 recognized subspecies, of which at least 9 have pathogenic potential. In Europe, most infections are caused by *B. afzelii, B. garinii*, and *B. burgdorferi* sensu stricto (s.s.) [3,4]. The list of animal hosts for Ixodid ticks that serve as reservoirs for *Borrelia* currently includes several hundred vertebrate species comprising mammals, reptiles, and birds [5]. A preventive vaccine is not yet available in human medicine, mainly due to the antigenic variability of the infectious agent [6].

Early infection is most often manifested by skin (various forms of erythema migrans) or/and flu-like symptoms (malaise, fatigue, headache, arthralgias, myalgias, fever, and regional lymphadenopathy). Without treatment, some patients may progress to later stages, most often affecting the nervous or musculoskeletal system, but the heart or eyes may also be affected [1].

In most cases, especially in the early stages, antibiotic treatment is highly effective but, in some patients, chronic problems such as myalgia, arthralgia, chronic fatigue, headaches, or cognitive deficits persist even after correctly indicated antimicrobial therapy. Various studies report a frequency of these complications in different proportions of Lyme disease patients, but the most common estimate is between 10 and 15% [7]. The term post-treatment Lyme disease syndrome (PTLDS) has been proposed for these conditions. Briefly, current guidelines define PTLDS as persistent or recurrent symptoms that began within 6 months of completion of a recommended antibiotic therapy for Lyme borreliosis and persist for 6 months or longer [8,9]. However, the exact definition and taxonomy of chronic clinical manifestations associated with Lyme disease are still the subject of discussions and polemics, and many less stringent definitions covering a broader group of patients have been published [10,11].

The diagnosis of PTLDS is based primarily on subjective symptoms and the method for objective confirmation is not available. PTLDS is thus usually diagnosed per exclusionem, by ruling out other possible pathological conditions. It is a matter of debate whether it is at all possible to make a diagnosis of PTLDS with final certainty.

The cause of the long-term symptoms has not yet been clarified; the involvement of immunopathological mechanisms associated with persistent inflammatory reaction and the formation of autoantibodies, the persistence of atypical morphological forms of bacteria or their residues, or the influence of other tick-borne infections are being intensively discussed [12,13].

Myositis-specific autoantibodies (MSA) are reported to be specific for inflammatory autoimmune skeletal muscle diseases, such as polymyositis and dermatomyositis. Recognized autoantigens are structurally and functionally diverse proteins, but many of them have in common the ability to interact with nucleic acids. The MSA usually includes antibodies against helicase Mi-2, chromatin-binding protein TIF-1, cytosolic double-stranded RNA sensor MDA-5, aminoacyl-tRNA synthetases (Jo-1, PL-7, PL-12, EJ, OJ), and nuclear matrix protein NXP-2. The other two autoantigens (SAE1, SRP) are physiologically involved in the translation and regulation of intracellular localization of proteins.

Myositis-associated autoantibodies (MAA) are found in addition to myositis also in other autoimmune disorders (systemic lupus, systemic sclerosis), or in overlapping syndromes (polymyositis/systemic sclerosis overlap syndrome). Autoantibodies against components of the RNA-processing exosome complex (PM-Scl75; PM-Scl100), DNA binding protein Ku, and ubiquitin ligase Ro-52 are usually included in MAA [14,15].

In our study, we investigated the occurrence of MSA, MAA, and anti-nuclear antibodies (ANA) in patients with suspicion of PTLDS with laboratory-proven history of Lyme disease. As the controls, we used a cohort of patients suspected of *Borrelia* infection as a part of differential diagnosis of neurological problems, such as paresis, paresthesia, or headache, in which laboratory history of Lyme disease was not confirmed and the final cause was determined to be demyelinating disorder affecting the central nervous system.

## 2. Materials and Methods

The study group included patients with a documented history of Lyme disease diagnosis followed by antibiotic therapy, at least in the time frame of current recommendations, who reported persistence of problems such as fatigue, musculo-articular pain, headache, and cognitive deficits after therapy completion. The anamnesis was evaluated on the basis of a patient’s interview with the clinicians of the Department of Immunology, the Department of Allergology and Clinical Immunology or the Third Department of Internal Medicine of the University Hospital Olomouc in accordance with published PTLDS definitions [8,9]. Blood sampling was also carried out in these departments. In both PTLDS and control cohorts, subjects diagnosed and treated for rheumatologic or other autoimmune disorders were excluded from the study. Individuals were referred to the study by co-authors of the study. Patients’ plasma was cultured as described in [16] with a negative result in all analyzed samples, examination of biopsy samples was not performed.

The control group represented the patients examined negative for Lyme disease by PCR and *Borrelia*-specific antibodies in the cerebrospinal fluid in the course of differential diagnosis of neurological disorders at the Department of Neurology of the University Hospital Olomouc. The examination was supplemented by determination of serum *Borrelia*-specific IgG antibodies. Patients with a negative result of all of *Borrelia*-specific tests with final diagnosis of demyelinating disorder of the multiple sclerosis type were included. Patients without definitive diagnosis, with other immunologically mediated comorbidities, or with a positive serological test for anti-*Borrelia* antibodies were excluded. The samples were taken during the diagnosis and therefore before immunosuppressive treatment was started. Samples were taken between May 2019 and April 2022.

The protocol of the study, including the informed consent of the patients, was approved by the Ethics Committee of the Olomouc University Hospital (reference number 102/18 of June 2018). Informed consent of all participating subjects was obtained.

The determination of parameters in cerebrospinal fluid was performed in an accredited clinical laboratory as a part of routine neurological laboratory investigation.

**Figure 1:**
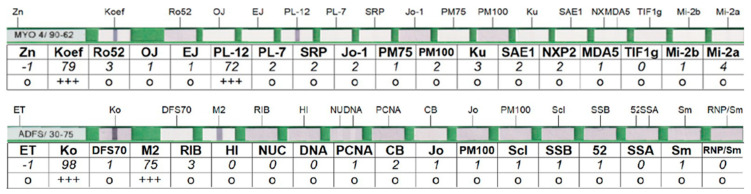
Examples of evaluated strips for the determination of myositis antibodies (**top**) and ANA antibodies (**bottom**).

Positivity of IgG antibodies against *Borrelia* in sera was confirmed using the Anti-*Borrelia* EUROLINE-RN-AT blot diagnostic kit (EUROIMMUN, Lübeck, Germany). The presence of autoantibodies was determined by immunoblot kit EUROLINE Autoimmune Inflammatory Myopathies 16 Ag (IgG) (EUROIMMUN, Luebeck, Germany) containing the following antigens: Mi-2a, Mi-2b, TIF1g, MDA5, NXP2, SAE1, Ku, PM-Scl100, PM-Scl75, Jo-1, SRP, PL-7, PL-12, EJ, OJ, Ro-52 and EUROLINE ANA profile 3 plus DFS70 (IgG) kit containing antigens nRNP/Sm, Sm, SS-A, SS-B, Scl-70, Jo-1, dsDNA, nucleosomes, histones, ribosomal P-protein, AMA M2, Ro-52, PM-Scl, CENP B, PCNA, and DFS70. All blot tests were evaluated by a flatbed scanner and software EUROLineScan Software 3.4 (EUROIMMUN, Lubeck, Germany). An example of the evaluated tests is shown in Figure 1.

Determination of creatine kinase (CK) and myoglobin levels as markers of muscle damage was performed using established accredited methods for routine diagnosis at the Department of Clinical Biochemistry, University Hospital Olomouc. Because there are no generally accepted standards for these markers, for the purpose of our study, we used the following values issued by the laboratory for University Hospital Olomouc clinicians as the upper limit of the normal range of CK in male 2.85 µkat/L, in female 2.42 µkat/L; myoglobin in male 72 µg/L, myoglobin in female 58 µg/L.

IBM SPSS Statistics version 23 statistical software (IBM, New York, NY, USA) was used for data analysis. The incidence of antibodies in the patient and control groups was compared using the Chi-square test and Fisher’s exact test, respectively. Student’s *t*-test was used to compare the age distribution of the subjects. Age normality was assessed using the Shapiro–Wilk test. All tests were performed at a significance level of 0.05.

## 3. Results

**Figure 2:**
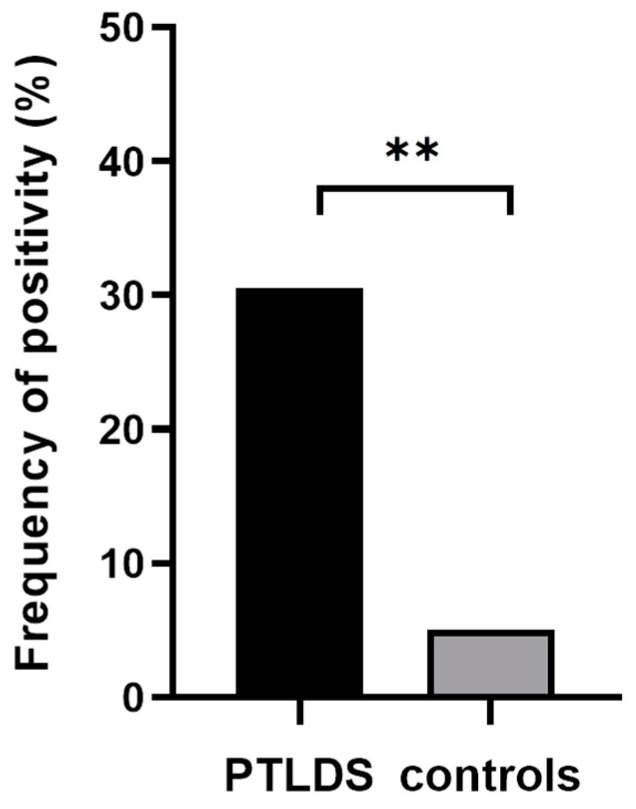
Comparison of the incidence of myositis autoantibodies in patients with suspected PTLDS (18/59) and control group (2/40). Indication of statistical significance: ** *p* < 0.01.

The positivity of at least one MSA or MAA was determined in 30.5% of patients with suspected PTLDS (18 positive samples from 59) in comparison to 5% of the subjects in the control group (2 positive samples from 40). Using Fisher’s exact test, the difference was evaluated as significant (*p* = 0.002, Figure 2).

Anti-Mi-2a autoantibodies were most frequently found in the PTLDS patients’ group (4/59; 6.8%), followed by anti-PL-7 and anti-PM/Scl-75 (both 3/59; 5.1%). Anti-Jo-1 anti-Ku and anti-PM/Scl-100 were each found in two patients (3.4%). Autoantibodies against Ro-52, PL-12, SRP, SAE1, MDA5, and Mi-2b were positive in only one patient each (1.7%). Four patients were positive for two antigens (4/59; 6.8%). In all of these four cases, Mi-2a/b was one of the responding antigens. Most positive reactions were rated as weak (+), in one case as medium (++), and in two cases as strong (+++), according to manufacturer recommendation (see material and methods). In the control group, myositis-related autoantibodies positivity was detected in only two subjects, of which one exhibited anti-PL-7 and one anti-Ku reaction. In both cases, it was a mild positivity (+).

For antigens also present in the ANA profile blot (Jo-1, Ro52, and PM-Scl), the identical result was obtained in all cases of positivity in both tests. With this ANA test, 5 PTLDS patients (8.6%) were found to be positive for anti-DFS70 antibodies, but these autoantibodies are described in similar frequencies in healthy subjects and no greater clinical significance is attributed to them [17]. One patient was found to be highly positive for anti-AMA M2 antibodies, which is antibody usually associated with liver diseases, but has recently been described as a marker of a new subtype of inflammatory myopathy [18]. In this patient, the SRP antigen was also positive and liver disease was ruled out in the laboratory (normal values of bilirubin, alkaline phosphatase, and aminotransferases). Furthermore, anti-Scl-70 positivity was recorded in two PTLDS patients and in one patient antibodies against histones were detected. In all cases, it was only a weak (+) reaction. Due to the low number of detected positivity of these antibodies with a different clinical association in the PTLDS group, these results were not meaningfully statistically comparable with the control group. The reaction with antigens nRNP, Sm, SS-A, SS-B, CENP B, PCNA, dsDNA, nucleosomes, and ribosomal P-protein was negative in all tested patients. The myositis autoantibodies positive results are shown in detail in Table 1.

Interestingly, none of the positive patients showed typical symptoms of myositis, such as muscle weakness limiting normal activities, dysphagia, or noticeable skin (Gottron’s sign) or lung involvement. Biochemical markers of muscle damage CK and myoglobin levels were determined in all patients with a positive result of the autoantibody test, except for one in which a sufficient volume of serum was not available for analysis. Values above the reference limit used in the University Hospital Olomouc were noted for CK in six patients and for myoglobin in three patients (Table 1). An increase in CK activity higher than twice the normal value was recorded in only one patient, and a more than twice increased concentration of myoglobin was also recorded in only one case. Even in patients with elevated CK activity, the increase was only modest and no clinical symptoms suggesting inflammatory myositis were detected.

All patients with elevated levels of muscle damage markers belong to the PTLDS group; in the control group, the results for CK and myoglobin in both myositis antibodies positive patients were within the physiological range.

## 4. Discussion

Individual case studies describing inflammatory muscle involvement in patients with Lyme disease of various duration have been available since the 1980s and 1990s [19]. Stainable spirochetes were demonstrated by histochemical methods in several muscle biopsies but culturing of *B. burgdorferi* was unsuccessful. Diagnosis of these “Lyme myositis” cases was mainly based on clinical and myopathological signs, given that specific detection of MSA and MAA was not yet available. However, more recent and comprehensive studies on this topic are very rare. Nilsson et al. [20] described positivity of myositis antibodies in 20% of 224 patients with persistent Lyme disease symptoms following tick bite, but the study did not include a control group without a history of health problems related to tick bite. Autoantibodies were found in 18–25% of patient groups seropositive for at least one tick-borne pathogen and 11% of seronegative individuals. However, the interpretation of the results is complicated by the fact that seronegative individuals were also included in the study based on problems assumed to be associated with tick borne infection, and more than 60% of individuals in this group underwent antibiotic treatment for that reason. In our study, we used for comparisons patients suffering from demyelinating disorders, which may exhibit similar symptoms to Lyme disease patients, but all control patients were negative in both serological and molecular biological routine tests proving history of *Borrelia* infection. The diagnosis of demyelinating disease of the multiple sclerosis type was confirmed by cerebrospinal fluid examinations and finally by magnetic resonance imaging. The determination of this final neurological diagnosis and the absence of anamnestic and laboratory proofs of *Borrelia* infection in patient´s records were used here to exclude the PTLDS in accordance with the definition. This may have influenced the fact that our result shows an even more significant difference in the frequency of autoantibodies between the observed groups.

Furthermore, the rate of positivity of MSA autoantibody in the present study does not correspond to previously published data about the specificity of MSA, such as anti-Mi-2, PL-7, and Jo-1, determined to be between 97.5 and 100% using Euroimmun line blot detection kits [21,22].

In previous publications, autoantibodies to various other antigens have been described in patients with complicated Lyme disease and PTLDS [13,23]. The broad spectrum of observed autoantibody specificities suggests that the induction of an immunopathological response is more probably due to the dysregulation of immunity in the sense of polyclonal activation, suppression of tolerance, or exposure of cryptic antigens during inflammatory process than previously thought cross-reactivity with *Borrelia* antigens associated with “molecular mimicry” [24]. In the case of cross-reactivity, a more uniform reaction with one or a narrow spectrum of structurally similar antigens would be expected.

In a recent study, elevated levels of myositis autoantibodies were also found in some patients hospitalized with COVID-19 [25]. This could suggest the involvement of some overlap in immunopathological mechanisms in the pathogenesis of PTLDS and long/post COVID symptoms.

## 5. Conclusions

Our data suggest the possibility of para- or post-infectious etiology of MSA/MAA in some cases of PTLDS in the absence of symptoms and biochemical findings typical for defined myositis, although these antibodies have so far been considered highly specific for biochemically and clinically confirmed inflammatory myopathies. In the group of MSA/MMA-positive patients, only less serious and non-specific manifestations such as fatigue, muscle pain, and reduced tolerance to physical exertion were observed. Although the levels of detected autoantibodies were mostly low and the patients did not fully develop symptoms of myositis, the high frequency of MSA/MAA antibodies support the hypothesis of the involvement of immunopathological mechanisms in the pathogenesis of PTLDS and could indicate a suitability of therapeutic approach in terms of temporary anti-inflammatory treatment or immunosuppression, rather than the prolonged or repeated antibiotic treatments still occasionally used today. However, we cannot rule out the possibility that some patients with suspicion of PTLDS may have a smoldering autoimmune disease manifested in a temporal connection with Lyme disease, but without a direct causal connection. Moreover, for this reason, detailed immunological laboratory examination and long-term follow-up is appropriate in patients with suspected PTLDS.

Our study, due to its design, is also not able to answer the question of whether the observed formation of autoantibodies is a sterile post-infection consequence of Lyme disease, or whether the autoantibodies are formed in association with the persistence of immunologically reactive *Borrelia* forms or their fragments.

## Figures and Tables

**Table 1:** Characterization of studied populations and results of determination of autoantibodies and biochemical markers. ANA antibodies shown in square brackets. Values of biochemical markers above the limit of normal values are shown with a gray background, results exceeding this limit twice or more are highlighted in bold. ND—not determined, CK—creatine kinase, MYO—myoglobin. Results were evaluated on a semi-quantitative scale: + weakly positive, ++ moderately positive, +++ strongly positive.

PTLDS Group				Control Group			
**Number of subjects**							
59				40			
**Average age; median of age; standard deviation**							
45.0; 46.0; 13.2				42.4; 39.5; 14.5			
**Gender ratio (Females/Males)**							
32/27				30/10			
**Myositis autoantibodies positive results**							
**Gender, age**	**Antigen positivity**	**CK (µkat/L)**	**MYO (µg/L)**	**Gender, age**	**Antigen positivity**	**CK (µkat/L)**	**MYO (µg/L)**
F, 28	MDA5 +	3.19	40	F, 27	PL-7 +	1.41	21.9
F, 54	PM-Scl75 +	1.95	54.1	M, 38	Ku +	2.25	70.3
M, 23	PL-7 +	ND	ND				
M, 57	PM-Scl100 +	1.52	523				
F, 47	Jo-1 + (Scl-70 +)	2.75	**198.7**				
F, 41	Jo-1 +	0.65	22.1				
M, 47	PL-12 +++	**6.14**	53				
F, 29	Mi-2a +; SAE1+	1.37	29.2				
M, 42	Mi-2a +	2.64	69,1				
F, 39	PM-Scl75 +	0.7	27.8				
M, 41	Ro-52 +++	3.21	115.1				
F, 22	PL-7 +; Mi-2b +	0.93	25.2				
M, 41	PM-Scl100 +	2.62	312				
M, 67	PM-Scl75 +	3.4	95.7				
F, 61	Mi-2a +; Ku +	1.32	32.6				
F, 57	Mi-2a +; PL-7 +	3.14	22.2				
M, 63	SRP ++ (AMA-M2 +++)	0.84	45.9				
F, 21	Ku +	1.36	34.1				

## Data Availability

The datasets used and/or analyzed during the current study are available from the corresponding author on reasonable request.
